# Experimental diabetes exacerbates autophagic flux impairment during myocardial I/R injury through calpain‐mediated cleavage of Atg5/LAMP2

**DOI:** 10.1111/jcmm.17642

**Published:** 2022-12-23

**Authors:** Lichun Guan, Ziqin Yu, Zhimei Che, Hang Zhang, Yong Yu, Dicheng Yang, Dewei Qian, Ruizhen Chen, Min Yu

**Affiliations:** ^1^ Department of Cardiovascular Surgery, Shanghai General Hospital Shanghai Jiao Tong University, School of Medicine Shanghai China; ^2^ Department of Cardiology, Shanghai Institute of Cardiovascular Diseases, Zhongshan Hospital Fudan University Shanghai China; ^3^ Department of Anesthesiology, Shanghai Chest Hospital Shanghai Jiao Tong University, School of Medicine Shanghai China; ^4^ Department of Cardiovascular Surgery, Nanjing First Hospital Nanjing Medical University Nanjing China

**Keywords:** Atg5, autophagic flux, calpain, diabetes, LAMP2, myocardial ischemia–reperfusion injury

## Abstract

To explore the role of autophagic flux in the increased susceptibility of the experimental diabetic heart to ischaemia‐reperfusion (I/R) injury, we established STZ‐induced diabetic mice and performed I/R. In vitro, neonatal mouse cardiomyocytes were subjected to high glucose and hypoxia/reoxygenation challenge to mimic diabetic I/R injury. We found that experimental diabetes aggravated I/R‐induced injury than compared with nondiabetic mice. Autophagic flux was impaired in I/R hearts, and the impairment was exacerbated in diabetic mice subjected to I/R with defective autophagosome formation and clearance. Calpains, calcium‐dependent thiol proteases, were upregulated and highly activated after I/R of diabetes, while calpain inhibition attenuated cardiac function and cell death and partially restored autophagic flux. The expression levels of Atg5 and LAMP2, two crucial autophagy‐related proteins, were significantly degraded in diabetic I/R hearts, alterations that were associated with calpain activation and could be reversed by calpain inhibition. Co‐overexpression of Atg5 and LAMP2 reduced myocardial injury and normalized autophagic flux. In conclusion, experimental diabetes exacerbates autophagic flux impairment of cardiomyocytes under I/R stress, resulting in worse I/R‐induced injury. Calpain activation and cleavage of Atg5 and LAMP2 at least partially account for the deterioration of autophagic flux impairment.

## 1 INTRODUCTION

Although revascularization is an important therapeutic measure to improve cardiac function and prognosis in patients with coronary heart disease, myocardial reperfusion therapy itself can also cause myocardial injury, known as myocardial ischaemia–reperfusion injury (MIRI).^1^ Diabetes mellitus, a rapidly growing global metabolic disease, increases susceptibility to MIRI and consequent adverse clinical outcomes.^2^ Hyperglycaemia potentiates the production of reactive oxygen species (ROS) of mitochondrial or nonmitochondrial sources and blunts antioxidant defence systems. Diabetes also induces impairment of many pro‐survival transduction pathways and abolishes the protective effect of ischaemic preconditioning.^3^, ^4^ Several of the mechanisms involved have been reported. For instance, diabetes activates Nox2‐related programmed cell death and aggravates MIRI.^5^

Diabetic adipocytes deliver small extracellular vesicles containing miR‐130b‐3p to cardiomyocytes, which suppress multiple anti‐apoptotic and cardioprotective factors and exacerbate MIRI.^6^ Nevertheless, the underlying mechanisms remain to be fully elucidated.

Autophagy is a highly conserved process of degradation of long‐lived cytoplasmic proteins and damaged organelles to meet intracellular metabolic demands and respond to extracellular stress. To date, autophagic flux is defined as the entire dynamic process of autophagy, including the formation of autophagosomes, the fusion of autophagosomes with lysosomes and the subsequent degradation and release of macromolecules into cytosol.^7^ Cardiomyocytes enhance autophagy to alleviate the energy crisis and remove injured mitochondria via adenosine monophosphate‐activated protein kinase (AMPK) activation during the ischaemia phase and Beclin 1 upregulation during reperfusion.^8^ Ma et al. reported that autophagic flux is partially impaired during reperfusion, and the recovery of autophagic flux can reduce the oxidative stress injury of cardiomyocytes and rescue MIRI.^9^, ^10^ However, whether autophagic flux impairment plays a role during MIRI in the context of diabetes is unclear.

Calpains are members of the calcium‐dependent thiol protease family. Calpain‐1 (μ‐calpain) and calpain‐2 (m‐calpain) are the main isoforms of calpains expressed in cardiomyocytes. Calpains are activated during reperfusion and contribute to MIRI through proteolysis of membrane cytoskeleton and myofibrillar proteins, modification of apoptosis‐inducing factors and promotion of excessive ROS.^11^, ^12^ Our previous research demonstrated that mitochondrial calpain‐1/2 can suppress mitochondrial fusion during reperfusion and lead to cell death.^13^ Calpains are involved in the autophagic process by cleaving numerous autophagy‐related proteins.^14^ For instance, calpain‐1/2 can cleave Atg5 into 24KD fragments, thereby inhibiting autophagy and inducing apoptosis in neutrophils and Jurkat cells.^15^ In the process of ischaemia/reperfusion (I/R) injury of retinal cells and hepatocytes, calpains have been discovered to inhibit autophagy through the cleavage of Beclin1 and Atg7.^16^, ^17^ In neuronal cells, calpain‐mediated cleavage of lysosome‐associated membrane protein 2(LAMP2) results in lysosome dysfunction and contributes to neuronal death.^18^ SQSTM1/P62, a marker of autophagic flux, is also a substrate of calpain.^19^ However, whether calpains affect autophagic flux during diabetic MIRI and its particular substrates has not been clarified.

In this study, we challenged streptozotocin(STZ)‐induced diabetic mice with myocardial I/R; and treated primary cardiomyocytes with high glucose (HG) and hypoxia/reoxygenation(H/R). We tried to clarify (i) whether autophagic flux impairment leads to increased susceptibility to MIRI in diabetic hearts; (ii) whether calpain activation plays a role in autophagic flux impairment of diabetes MIRI; (iii) and whether calpain regulates autophagic flux via degradation of autophagy‐related proteins during diabetic MIRI.

## 2 MATERIALS AND METHODS

### 2.1 Animals

Male inbred C57BL/6 mice aging 6–8 weeks were used for the experiment. The transgenic calpastatin‐overexpression mouse strain (Tg‐CAST) was provided by the laboratory of Tianqing Peng (Lawson Health Research Institute, Canada).^13^ All animal experiments were approved by the Ethics Review Board for Animal Studies of Shanghai Jiao Tong University School of Medicine (approval no. SYKX‐2008‐0050) and were conducted in strict accordance with the Guide for the Care and Use of Laboratory Animals published by the US National Institutes of Health (NIH Publication, 8th Edition, 2011).

### 2.2 Induction of diabetes and experimental protocol

Diabetes was induced in overnight fasted mice by administering a single intraperitoneal (ip) injection of 50 mg/kg streptozotocin (STZ) dissolved freshly in 0.1 mol/L sodium citrate buffer (pH 4.5). The control group (Con) mice were injected with a similar volume (100 μl) of sodium citrate buffer alone. The mice with fasting blood glucose higher than 16.7 mmol/L after 5 consecutive days of injection (50 mg/kg STZ for each injection) were considered diabetic (Figure S1K). All mice were fed for 1 month before surgery.

### 2.3 Identification of transgenic mice

All Tg‐CAST littermates were genotyped by DNA electrophoresis following PENG's lab protocol as previously described^13^ (Figure S1A).

### 2.4 Myocardial ischemia/reperfusion model establishment

Mice were anaesthetized with 2% isoflurane inhalation on an isoflurane delivery system (Viking Medical) without ventilation. Then, we made a 1 cm incision in the left fourth intercostal space. The left coronary artery (LCA) was ligated (no ligation for sham operation group) for 30 min followed by 12 h of reperfusion as previously described.^13^ At the indicated time points, the mice were sacrificed by cervical dislocation, and the hearts were immediately extracted for further analysis.

### 2.5 Myocardial infarct size measurement

Heart slices were stained using Evans blue (Sigma, 17779, E2129) and 2, 3, 5‐triphenyl tetrazolium chloride (TTC), and myocardial infarct size and area at risk was measured by ImageJ 1.37 software.

### 2.6 Echocardiography

Echocardiography was conducted by M‐mode echocardiography using a Philips IE33 instrument (Philips Medical Systems Corporation) with a 1–5 MHz transducer (S5‐1), under the instruction of the guideline. Two‐dimensional‐guided M‐mode measurements of the LV internal diameter were obtained from the short‐axis view at the papillary muscles' level over at least three beats and were averaged.

### 2.7 Transmission electron microscopy (TEM)

Cardiac tissue from mouse was fixed for TEM as previously described.^13^

### 2.8 Serum cTnI detection

Serum cTnI level was detected by an ELISA kit (ab188877, Abcam) following the manufacturer's instructions to evaluate myocardial I/R injury.

### 2.9 Cardiomyocyte culture and induction of hypoxia/reoxygenation (H/R) based on high‐glucose (HG)

Neonatal mouse (1 day old, Department of Laboratory Animal Science, Fudan University) ventricular cardiomyocytes were isolated and cultured as previously described with a few modifications.^13^

### 2.10 Cardiomyocyte viability assay

Cultured cardiomyocyte viability was assessed by Cell Counting Kit‐8 (C0038, Beyotime Biotechnology) as previously described.^13^ In brief, the ventricles were enzymatically digested in 0.08% collagenase II. After centrifugation and resuspension, cells were plated in culture dishes and incubated at 37°C in a 5% CO2 incubator by DMEM with a glucose concentration of 5.5 mmol/L (control group, NG) and 33 mmol/L (high glucose, HG group) for 48 h. To rule out the effect of hyperosmolarity, 33 mmol/L mannitol was checked for the impact on viability, calpain activity and autophagic activity, and the results showed hyperosmolarity did not affect our results (Figure S1B‐D). To be treated with hypoxia/reoxygenation(H/R) process, the isolated myocardial cells were moved to a hypoxia incubator (1% O2, 94% N2 and 5% CO2) for 12 h, followed by reoxygenation in normoxic conditions for 3 h at 37°C.

### 2.11 Western blot analysis

The area of I/R injury in the heart was collected in an EP tube with RIPA protein lysis buffer containing protease inhibitors. Protein concentrations were measured with a BCA assay kit (P0010, Beyotime Biotechnology). The proteins obtained above were separated by 10%–15% SDS‐PAGE and then transferred onto a polyvinylidene difluoride (PVDF, Millipore) membrane, placed in 5% bovine serum albumin (BSA, QIANTU BIO) containing TBST at room temperature for 1 h and then incubated overnight at 4°C with primary antibody diluted in TBST with 5% BSA. Primary antibodies include the following: LC3 (1:1000, 4108, CST), P62 (1:1000, 23,214, CST), calpain‐1 (1:1000, 2556, CST), calpain‐2 (1:1000, 2539, CST), Beclin‐1 (1:1000, 3495, CST), Atg5 (1:1000, 2630, CST), LAMP2 (1:1000, ab13524, Abcam), β‐ACTIN (1:1000, 3700, CST). The PVDF membranes were washed and then incubated with HRP‐conjugated secondary antibodies at room temperature for 1 h. An ECL‐HRP chemiluminescence kit (34,577, Thermo Fisher) was used to visualize the bands, which were quantified by Image Lab Software (Bio‐Rad). All samples were analysed in triplicate with a multilabel reader (excitation, 360 nm; emission, 460 nm, Thermo).

### 2.12 Immunohistochemical staining

Briefly, heart tissues were embedded in paraffin and cut into 10 μm slices. The slices were incubated at 4°C overnight with a 1:50 dilution of anti‐LC3II (1:1000, 3868, CST) or SQSTM1/P62 antibody and then incubated with a biotinylated goat anti‐rabbit secondary antibody (1:200, ab150077, Abcam) for 45 min. The samples were added tri‐antibody (SAB complex) for 40 min. The samples were observed by DAB chromogenic method, and the particular brown strength was taken as positive and was counted by ImageJ Software.

### 2.13 Calpain activity detection

A fluorometric calpain activity assay kit (ab65308, Abcam) was used to quantify calpain activity in the lysates of myocardial cells or heart tissue by fluorescent substrate N‐succinyl‐LLVY‐AMC. All samples were analysed in triplicate with a multilabel reader (excitation, 360 nm; emission, 460 nm, Thermo, America) and expressed as relative fluorescent units (RFU) as previously described.^13^

### 2.14 Quantitative real‐time reverse transcription PCR (qRT‐PCR)

Total RNAs were extracted from primary cardiomyocytes using TRIzol Reagent (Invitrogen; 15,596,018). 500 ng RNA per sample was used for reverse transcription and qRT‐PCR assay. The qRT‐PCRs were performed using SYBR Premix Ex Taq (TaKaRa; Cat. No. RR420A) according to the manufacturer's instructions on an ABI Prism 7500 Real‐Time PCR System (Applied Biosystems). β‐actin was used as the internal control. The data were analysed by using the comparative CT method. The primers used in this study for qRT‐PCR were as follows:

β‐actin.

Forward: 5’‐AGAGCCTCGCCTTTGCCGAT‐3′.

Reverse: 5′‐ TGCCAGATTTTCTCCATGTCGT ‐3′.

ATG5.

Forward: 5’‐GGACCTTCTACACTGTCCATCC‐3′.

Reverse: 5’‐TGTCATTCTGCAGTCCCATC‐3′.

LAMP2.

Forward: 5′‐CTAGGAGCCGTTCAGTCCAA‐3′.

Reverse: 5′‐CTTGCAGGTGAATACCCCAA‐3′

### 2.15 Adenoviral transfection

To overexpress Atg5 and LAMP2, Ad‐vector, Ad‐ATG5 and Ad‐LAMP2 were synthesized by HanBio, China. Cardiomyocytes were infected with each of the three adenoviruses. An empty adenovirus (Ad‐vector) served as a control at a multiplicity of 100 PFU/cell infection. 48 h after cell plating, serum was removed and the cells were infected with recombinant adenovirus. Successful transfection was verified by WB‐analysis (Figure S1E,F). For in vivo studies, adenoviruses were expressed in the heart by myocardial in situ injection at a titre of 4 × 10^10^ PFU/ mice. Exposing the mouse heart (the method is the same as the establishment of I/R model), a 10 μl viral suspension was injected into the myocardium at four points around the heart's coronary arteries by a microsyringe. After 3 days, mice were subjected to I/R (Figure S1G). Successful transfection was verified by fluorescent images (Figure S1H).

### 2.16 Measurement of fluorescent LC3 puncta

Cultured cardiomyocytes were transfected with Ad‐mCherry‐GFP‐LC3 (adenovirus expressing mCherry‐GFP‐LC3B fusion protein, HanBio Technology, China, MOI = 100, *n* = 30) following the manufacturer's instructions. After treatment with HG and/or HR, cells were fixed with 4% paraformaldehyde and observed by an Olympus confocal microscope (Olympus). Yellow puncta and red‐only puncta in merged images were counted by ImageJ Software.

### 2.17 Immunofluorescence staining

Cultured cardiomyocytes were fixed with 4% paraformaldehyde after various treatments. After being blocked with 5% BSA, the rabbit anti‐mouse ATG5 (1:100, 2630, CST) and Lamp2 (1:100, ab13524, Abcam) antibodies were incubated with the cells overnight at 4°C. Cy3‐labelled goat antirabbit second antibody (1:50) was incubated for 2 h at 37°C in the dark. Antifade Mounting Medium with DAPI (4,6‐Diamidino‐2‐phenylindole, Beyotime, P0131) was added to incubate for 10 min. Cells were captured by an Olympus confocal microscope (Olympus), and the fluorescence intensity was analysed with ImageJ software.

### 2.18 Statistical analysis

All statistical tests were analysed with GraphPad Prism 6.02. All data were expressed as means ± SD. Rank sum test was used for comparisons between two groups, and one‐way analysis of variance (anova) was used for comparisons of three or more groups, followed by the Bonferroni post hoc test. *p* < 0.05 was considered statistically significant.

## 3 RESULTS

### 3.1 Autophagic flux correlates with susceptibility to MIRI in STZ‐induced diabetic mice

**FIGURE 1:**
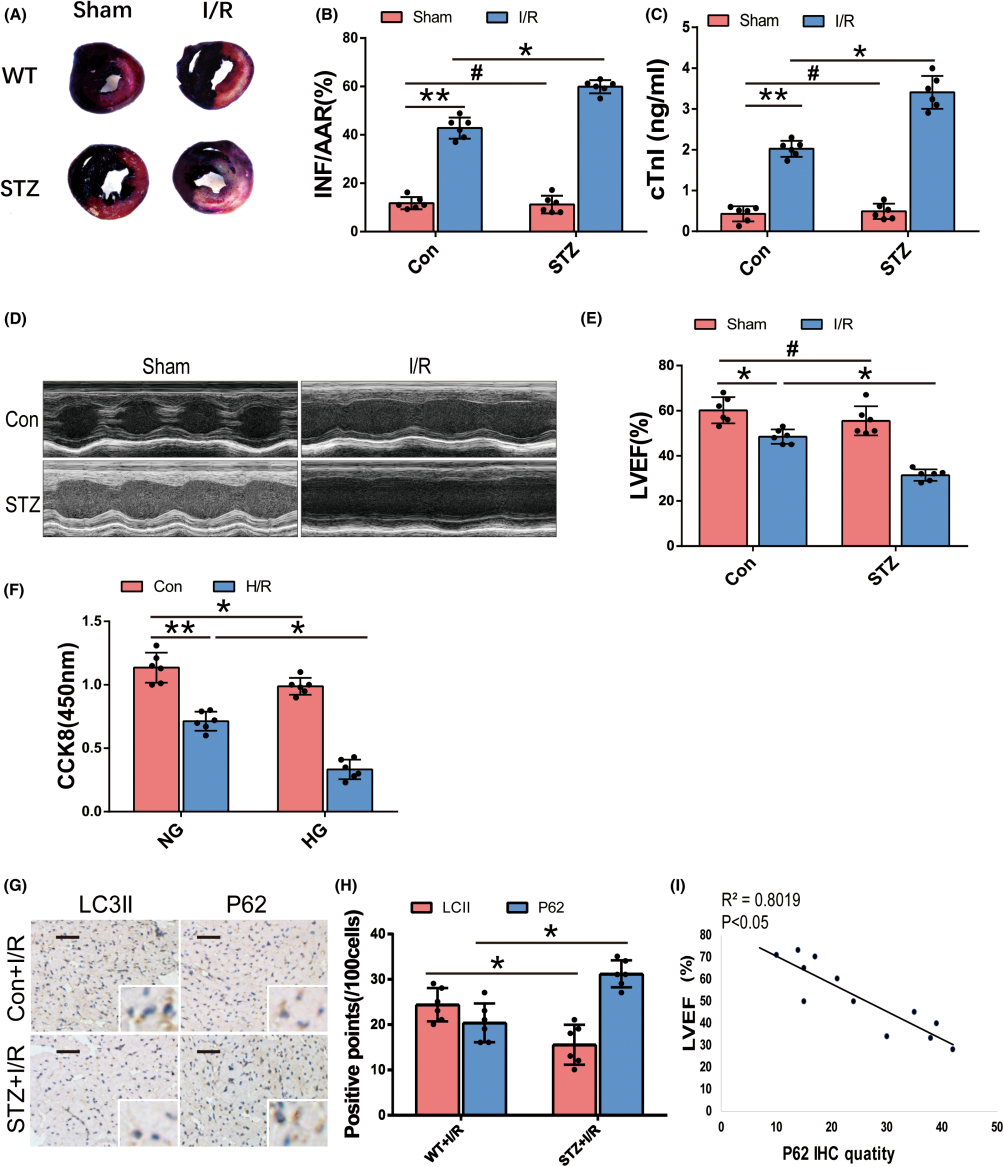
Experimental diabetic MIRI in vivo and in vitro, and the association with autophagy‐related markers. (A) Representative images of Evans blue‐TTC staining of heart samples which showed myocardial infarction (white), area at risk (red) and perfused area (blue) of Con+Sham, STZ + Sham, Con+I/R and STZ + I/R group (*n* = 6, each group). (B) Quantitative analyses of the ratio of infarction and risk area (INF/AAR%) of the four groups. (C) Serum cTnI by ELISA of the four groups. (D,E) Representative images of cardiac function by echocardiography and analysis of LVEF of the four groups. (F) Cell viability of cultured neonatal mouse cardiomyocytes by CCK8 assay of Con, HG, H/R and HG + H/R group (*n* = 6, each group). (G,H) Representative images of immunohistochemical staining of P62 and LC3II (brown dots) in heart samples of Con+I/R group and STZ + I/R group after 12 h of reperfusion (*n* = 6, each group) and quantification analysis (*n* = 100 cells from 6 individual experiments, scale bar = 50 μm). The magnification is 100 and 400 times. (I) Linear correlation between quantity of myocardial P62 expression (positive points per 100 cells) and LVEF value in Con+I/R and STZ + I/R mice. (*p < 0.05; **p < 0.01; ^#^ *p* > 0.05).

To determine the effect of diabetes on MIRI, we assessed myocardial infarct and heart function by Evans blue/TTC staining, serum cTnI and echocardiography in the mouse groups. The STZ‐induced diabetic mice subjected to I/R (STZ + I/R group) showed a larger infarction area, a higher serum cTnI and a lower LVEF than nondiabetic control mice subjected to I/R (Con+I/R group) (Figure 1A‐E). STZ‐induced diabetes alone significantly affected the myocardial infarction area, cTnI and LVEF in our model, as we employed a short period of intervention (Figure 1A‐E). Other echocardiographic indices including FS, LVEDD and LVESD are shown in (Figure S1L). Consistently, in vitro, neonatal cardiomyocytes were treated with HG followed by H/R stress (HG + H/R group) to mimic diabetic I/R injury. The loss of viability measured by CCK8 assay was mild under HG conditions and predominant under H/R stress. Cell viability was lowest in the HG + H/R group (Figure 1F). These results suggest that experimental diabetes exacerbates MIRI in vitro and in vivo. Then, we tested the expression of the autophagy‐related markers LC3II and P62, which represent autophagosome formation and clearance,^7^ by immunohistochemical (IHC) staining in heart samples in the Con+I/R and STZ + I/R groups. Compared to Con+I/R groups, cardiomyocytes in the STZ + I/R group expressed significantly lower levels of LC3II, but higher levels of P62 (Figure 1G,H). Interestingly, we found that the expression of P62 was inversely correlated with the echocardiographic LVEF values (*R* ^2^ = 0.8292, *p* < 0.05) (Figure 1I). These results indicate that reduced LC3II and increased P62 expression are associated with excessive MIRI in diabetes.

### 3.2 Experimental diabetes exacerbates I/R‐induced autophagic flux Impairment

**FIGURE 2:**
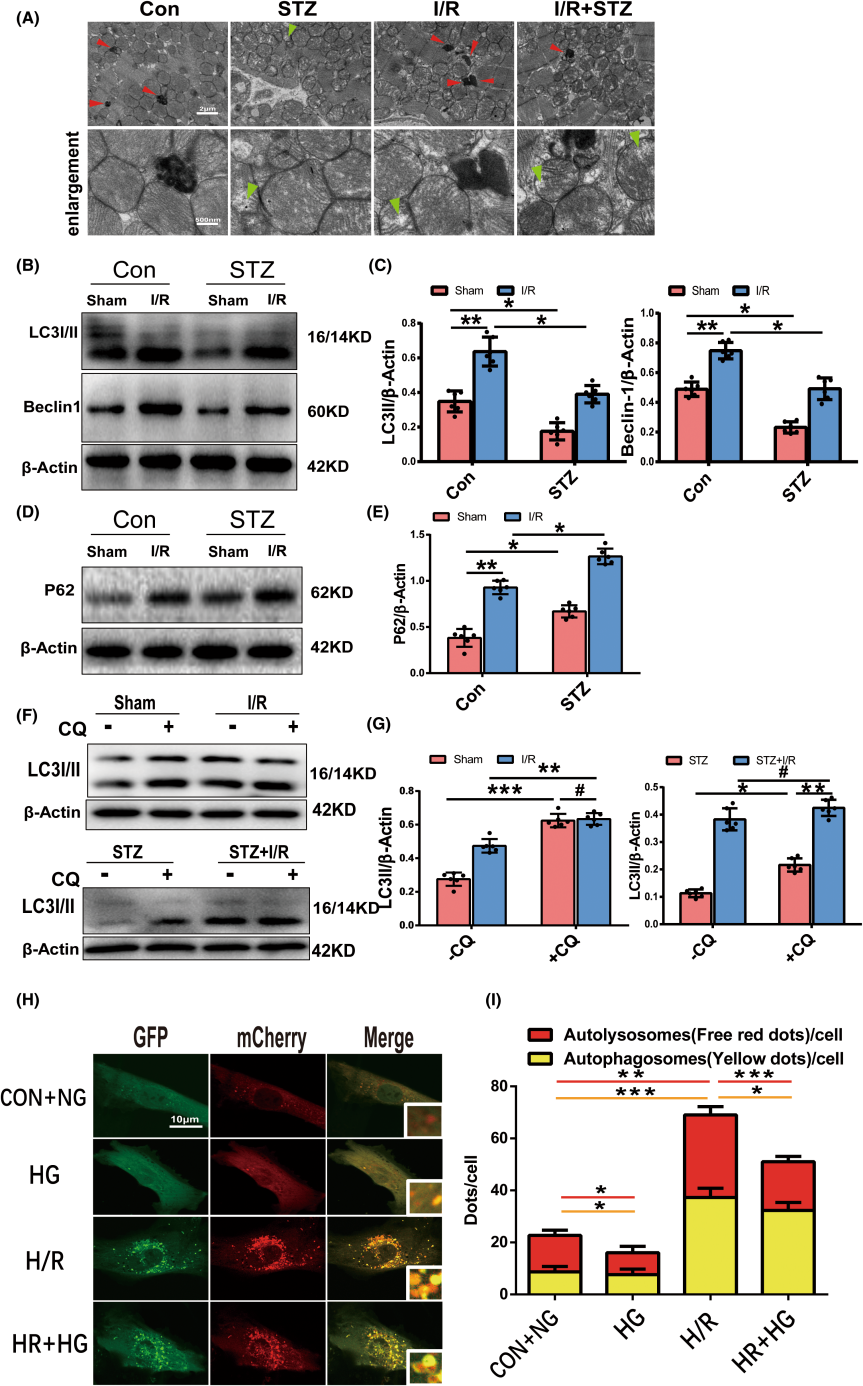
Autophagic flux during diabetic MIRI. (A) Representative illustrations of TEM showed autophagosome and mitochondrial ultrastructural in each group. The quantity of autophagosomes (red arrow) indicated dynamic autophagic flux; and mitochondrial vacuolization and cristae disorder (green arrow) indicated the extent of mitochondrial damage. (Scale bar = 2 μm,500 nm) (B,C) Western blot and quantitative analyses of LC3II and Beclin1 expression in hearts of each group (*n* = 6 per group). (D,E) P62 expression by Western blot and quantitative analyses. (F,G) LC3 expression with or without CQ intervention (50 mg/kg injection intraperitoneally, half an hour before surgery) in each group by Western blot and quantitative analyses. (H,I) Representative images of laser confocal microscopy of mCherry‐GFP‐LC3 puncta in cultured mice cardiomyocytes of the four groups and quantitative analyses of autophagosomes (yellow dots) and autolysosomes (red dots) per cell. (Scale bar = 10 μm, *n* = 30 cells per group). *p < 0.05; **p < 0.01; ***p < 0.001; ^#^ *p* > 0.05).

To clarify the impact of diabetes and/or I/R on autophagic flux, we assessed the autophagosome morphology by TEM and autophagy‐related markers (LC3II, Beclin1 and P62) by Western blot in the mouse model. TEM showed that a certain number of autophagosomes were formed in the control mice, accompanied by neat muscle fibres and intact dense mitochondria. However, the quantity of autophagosomes in STZ‐induced diabetic mice was minimal, and a few mitochondrial vacuoles were observed. I/R treatment in nondiabetic mice caused the accumulation of autophagosomes, and mitochondria became vacuolated with disordered crista. Compared to I/R mice, STZ + I/R mice showed fewer autophagosomes and more pronounced damage in the mitochondrial morphology (Figure 2A). In accordance with TEM, Western blotting revealed that LC3II and Beclin1 were downregulated in STZ‐induced diabetic sham mice and upregulated in I/R mice compared with the control sham group. Compared to Con+I/R mice, LC3II and Beclin1 expression levels were lower in STZ + I/R mice (Figure 2B,C). However, myocardial P62 expression was elevated in mice subjected to STZ or I/R and highest in the STZ + I/R group, which indicated that there were defects in autophagosome clearance in the three groups. (Figure 2D,E).

Then, we used chloroquine (CQ), a late autophagy inhibitor that blocks the fusion of autophagosomes and lysosomes and the degradation of autolysosomes, to evaluate autophagic flux; CQ intervention induced a significant increase in LC3II expression in the Con+Sham group. The CQ‐induced increase in LC3II expression in the I/R group was similar to that in the Con+Sham group, indicating that autophagic flux is partially blocked during nondiabetic I/R. Nevertheless, LC3II expression was only slightly altered under CQ intervention in the STZ + I/R group, which indicated that autophagic flux was completely blocked during diabetic I/R stress (Figure 2F,G).

To fully evaluate autophagic flux in vitro, cardiomyocytes were transfected with Ad‐mCherry‐GFP‐LC3. There were more autolysosomes (red dots) than autophagosomes (yellow dots) in control cells. The numbers of autolysosomes and autophagosomes were clearly decreased following HG, indicating less autophagosome formation under HG treatment, in agreement with the results of TEM and autophagy‐related protein expression. H/R stress resulted in dramatic increases in autophagosomes and autolysosomes, but the autolysosome‐to‐autophagosome ratio was reversed, indicating that H/R stimulates autophagosome formation and hinders clearance. When cardiomyocytes were subjected to H/R following HG, the increase in autophagosomes was less than that after H/R treatment alone, with a similar number of autolysosomes to the control group and a low autolysosome‐to‐autophagosome ratio (Figure 2H,I). Taken together, these data show that experimental diabetes inhibits autophagy induction in cardiomyocytes and that I/R induces autophagy and partially impairs autophagosome clearance. During diabetic I/R injury, autophagic flux is worse than that during nondiabetic I/R injury, with impairments of both autophagosome formation and clearance.

### 3.3 Autophagic flux affects vulnerability to I/R injury in the diabetic heart

**FIGURE 3:**
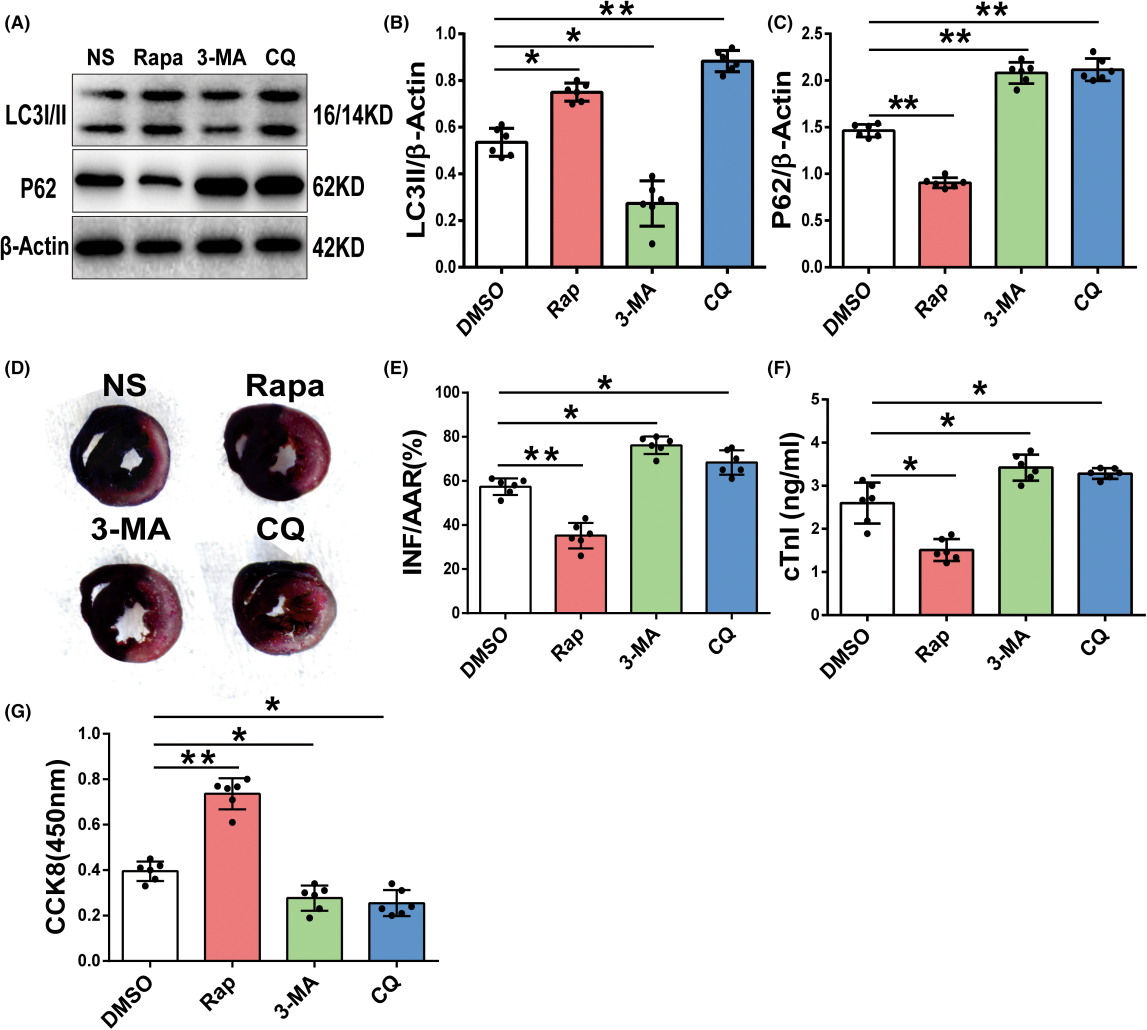
The role of autophagic flux in diabetic MIRI. (A‐C) LC3 and P62 expression by Western blot of diabetic mice subjected to I/R with NS, rapamycin (1 mg/kg), 3‐MA (30 mg/kg) or CQ (60 mg/kg) injection intraperitoneally 0.5 hours before I/R treatment and quantitative analyses (*n* = 6 per group). (D,E) Representative images of Evans blue‐TTC staining of heart samples of the four groups and quantitative analyses of ratio of myocardial infarction size to area at risk. (F) Serum cTnI level by ELISA of the four groups. (G) Cell viability of cultured mice cardiomyocytes after HG and H/R stress pretreated with DMSO, rapamycin (0.2 μM), 3‐MA (5 mM) or CQ (10 μM) by CCK8 assay (*n* = 6 per group; *p < 0.05; **p < 0.01).

To determine the role of autophagic flux in diabetic MIRI, we modulated autophagic flux in the STZ + I/R mice with (i) 0.9% normal saline (NS); (ii) rapamycin (Rapa, 1 mg/kg), an inhibitor of mTOR, to promote autophagic flux; (iii) 3‐methyladenine (3‐MA, 30 mg/kg), an inhibitor of type III phosphatidylinositol 3‐kinases (PI3K), to suppress autophagosome formation; and (iv) CQ (50 mg/kg) to block the fusion of autophagosomes and lysosomes and degradation, (intraperitoneal injection, half an hours before I/R treatment). Rapamycin treatment induced higher LC3II and lower P62 expression levels, which indicated that Rapa promoted the autophagic flux of diabetic hearts subjected to I/R (Figure 3A‐C).Rapamycin significantly decreased the areas of myocardial infarction and the serum cTnI levels compared with NS treatment (Figure 3D‐F). 3‐MA downregulated LC3II expression, and CQ increase it, while both increased P62 expression (Figure 3A‐C). 3‐MA and CQ impaired autophagic flux, and both enlarged the infarct size and cTnI release of diabetic I/R mice (Figure 3D‐F). Consistently, in cultured cardiomyocytes subjected to HG + H/R, the CCK8 assay also showed higher cell viability in the rapamycin (0.1 μM) treatment group than in the DMSO treatment group, and lower cell viability in the autophagic flux inhibitor 3‐MA (5 mM) and CQ (10 μM) treatment groups (Figure 3G). These results indicate that autophagic flux plays a vital role in MIRI of the diabetic heart and that enhanced autophagic flux attenuates diabetic MIRI, while impaired autophagic flux exacerbates MIRI.

### 3.4 Calpain‐1 and calpain‐2 activation contributes to autophagic flux impairment and diabetic MIRI

**FIGURE 4:**
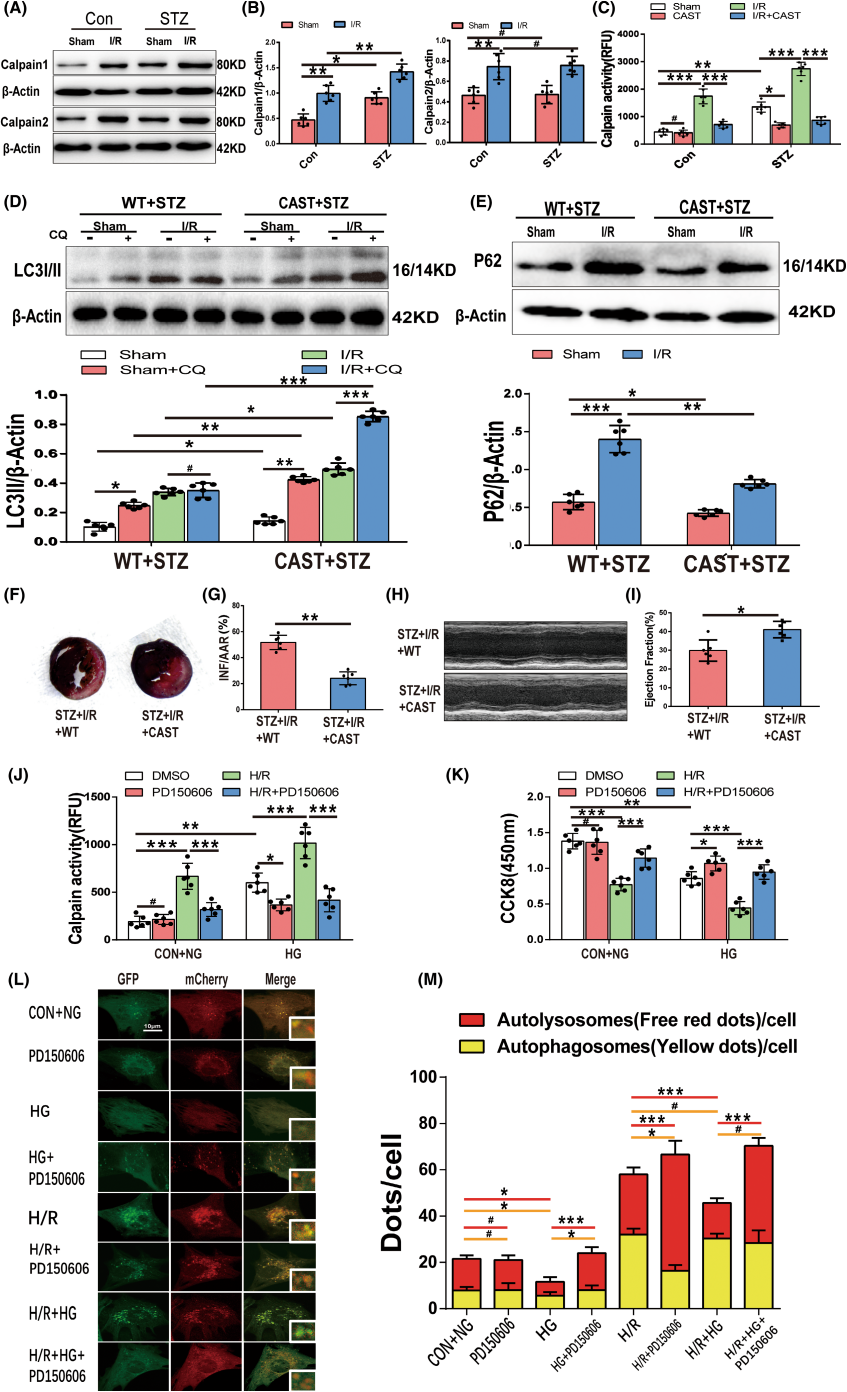
Calpain activation is involved in autophagic flux impairment and diabetic MIRI. (A,B) Cardiac calpain‐1 and calpain‐2 expression during SZT‐induced diabetes, I/R and diabetic I/R by Western blotting and quantitative analyses (*n* = 6 per group). (C) Calpain activity by N‐succinyl‐LLVY‐AMC fluorometry of the four wild‐type mice groups and corresponding Tg‐CAST mice groups. (D) Relative LC3II expression by Western blot and quantitative analyses in wild‐type mice and Tg‐CAST mice under STZ and I/R stress with or without CQ treatment. (E) P62 expression by Western blot and quantitative analyses. (F,G) Representative images of Evans blue‐TTC staining of heart samples of WT mice and Tg‐CAST mice with diabetic I/R and quantitative analyses of ratio of myocardial infarction to area at risk (*n* = 6 per group). (H,I) Representative images of echocardiography of WT mice and Tg‐CAST mice with diabetic I/R and analyses of LVEF. (J) Calpain activity of cultured cardiomyocytes under H/R and(or) H/R stress with or without PD150506 treatment (*n* = 6 per group). (K) Viability of cultured cardiomyocytes by CCK8. (L,M) Representative images of laser confocal microscopy of mCherry‐GFP‐LC3 puncta in cultured mice cardiomyocytes of the eight groups and quantitative analyses of autophagosomes (yellow dots) and autolysosomes (red dots) per cell. (Scale bar = 10 μm, *n* = 30 cells per group). *p < 0.05; **p < 0.01; ***p < 0.001; ^#^ *p* > 0.05.

To determine the impact of calpains on autophagic flux impairment and diabetic MIRI, calpain expression and activity were assayed, as well as LC3II and P62 expression with and without calpain inhibition. Calpain‐1 expression was upregulated in the STZ‐induced diabetic heart compared to control mice, whereas calpain‐2 expression remained at the same level as control. However, both calpain‐1 and calpain‐2 expression levels were upregulated under I/R stress and showed more pronounced elevation in STZ + I/R mice (Figure 4A,B). Meanwhile, calpain activity was increased in the STZ and I/R groups, and further enhanced in STZ + I/R mice (Figure 4C).

**FIGURE 5:**
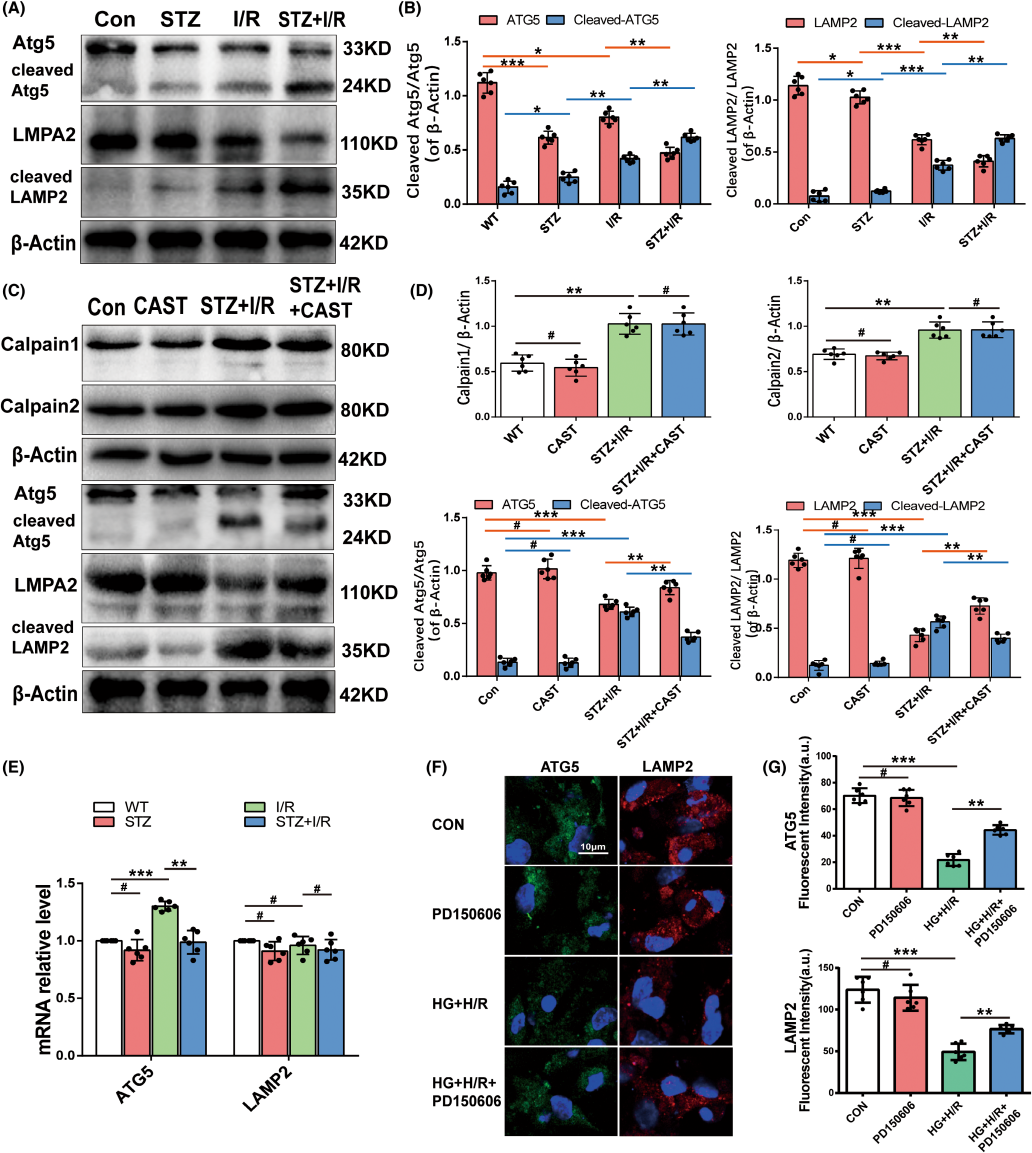
Calpain‐mediated downregulation of Atg5 and LAMP2 during diabetic MIRI. (A,B) Expression levels (corrected by β‐Actin level) of LAMP2 and Atg5 and their cleaved fragments during SZT‐induced diabetes, I/R and diabetic I/R by Western blotting and quantitative analyses. (C,D) Expression levels (corrected by β‐Actin level) of calpain‐1, calpain‐2, LAMP2, Atg5 and their cleaved fragments of wild‐type and Tg‐CAST mice underwent STZ plus I/R stress by Western blotting and quantitative analyses. (E) mRNAs levels of Atg5 and LAMP2 by RT‐qPCR of wild‐type mice hearts subjected to various treatment. (F,G) Representative images of LAMP2 (red) and Atg5 (green) expression of cultured cardiomyocytes subjected to HG and H/R with and without calpain inhibitor PD150606 by immunofluorescent staining (nuclei were located by DAPI (blue), Fluorescence 525/600 nm, arbitrary units, scale bar = 20 μm). and quantitative analysis (n = 30 cells per group). *p < 0.05; **p < 0.01; ***p < 0.001; ^#^ *p* > 0.05. (*n* = 6 per group).

Calpastatin is a specific endogenous calpain‐1/2 inhibitor. When calpains were activated under STZ or I/R stress, Tg‐CAST mice that overexpressed calpastatin inhibited calpain activity with no effect on calpain expression (Figures 4C and 5C), as we previously reported13. Wild‐type mice (WT) and Tg‐CAST mice (CAST) were subjected to STZ treatment and then to sham operation or I/R procedures. LC3II expression in the CAST STZ + Sham mice was upregulated compared to that in the WT STZ + Sham mice, and the CQ‐induced LC3II increase in the CAST STZ + Sham group was also higher than that in the WT STZ + Sham group indicating that calpain inhibition may alleviate the STZ‐induced autophagy suppression. LC3II expression in the CAST STZ + I/R group was slightly higher than that in the WT STZ + I/R group, with a marked additive increase under CQ intervention (Figure 4D). CAST also attenuated P62 accumulation under STZ or STZ + I/R stress (Figure 4E). Calpain inhibition restored autophagic flux in STZ + I/R hearts. CAST mice could resist the negative effect of diabetic I/R on the myocardium, exhibiting a smaller myocardial infarction area and a higher LVEF value (Figure 4F‐I).

PD150606 is a specific inhibitor of calpain activity. In vitro, cultured mouse cardiomyocytes pretreated with PD150606 presented less calpain activity and correspondingly higher viability under HG, H/R and HG + H/R stress (Figure 4J,K). In mCherry‐GFP‐LC3 fluorescent images, PD150606 pretreatment did not change the fluorescence intensity of control cells, but elevated autophagosomes and autolysosomes in the HG group to a nearly normalized state. Moreover, calpain inhibition relieved H/R‐induced autophagosome accumulation and increased the number of autolysosomes. During H/R plus HG stress, inhibition of calpain only predominantly increased autolysosomes and recovered the autolysosome‐to‐autophagosome ratio (Figure 4L,M). Taken together, these data indicate that calpain activation is involved in autophagic flux impairment of diabetic MIRI and that calpain inhibition restores autophagic flux by facilitating both autophagosome formation and clearance and rescuing cardiomyocytes.

### 3.5 Atg5 and LAMP2 are downregulated by activated calpains during I/R in experimental diabetic hearts

We then explored the underlying mechanism of calpain‐mediated autophagic flux impairment during diabetic MIRI. Several autophagy‐related proteins (Atg5, Atg7, Beclin1, P62 and LAMP2) have been reported to be substrates of calpains in other cells. Among them, the expression profiles of Atg7 and Beclin1 showed the same trends as LC3, which cannot be degraded by calpains (Figure S1I), and p62 was independent of calpain activity (Figure 4 E), so we excluded them as calpain targets. Atg5 and LAMP2 were highly expressed in control mice, and both diabetes and I/R alone resulted in LAMP2 and Atg5 reduction. I/R stress in the diabetic heart further decreased the levels of these two proteins. Notably, Atg5 expression was higher in the I/R group than in the STZ group, while LAMP2 appeared to be differentially expressed (Figure 5A,B). Cleaved fragments of Atg5(24KD) and LAMP2(35KD) were detected, which were minimal in control mice and gradually increased with STZ, I/R and STZ + I/R treatment (Figure 5A,B). We also checked the mRNA levels of LAMP2 and Atg5 by qRT–PCR. The mRNA levels did not change significantly in most situations, except that Atg5 transcription showed a significant elevation after I/R treatment (Figure 5E). Calpain can degrade Atg5 into24KD fragment and LAMP2 into a 35KD fragment15,18. As we found that calpain activity gradually increased after STZ, I/R and STZ + I/R stress, we concluded that the decreases in Atg5 and LAMP2 and the presence of cleavage products were due to calpain activation.

Then, we studied the protein expression of Atg5 and LAMP2 in Tg‐CAST mice that underwent STZ and I/R treatment. When calpain activity was inhibited by calpastatin overexpression, Atg5 and LAMP2 expression levels partially recovered, accompanied by a reduction in cleaved fragments (Figure 5C,D). These data also support that calpain upregulation and activation are responsible for Atg5 and LAMP2 downregulation.

In vitro, we examined Atg5 and LAMP2 expression by immunofluorescence. Consistently, the immunofluorescence intensities of LAMP2 (red dots) and Atg5 (green dots) were decreased in cultured HG + H/R cardiomyocytes. Treatment with the calpain inhibitor PD150606 reversed these reductions (Figure 5F,G). Taken together, we deduced that calpains are highly activated in cardiomyocytes of diabetic I/R mice and degrade Atg5 and LAMP2.

### 3.6 Co‐overexpression of LAMP2 and Atg5 rescues autophagic flux and viability of diabetic MIRI

**FIGURE 6:**
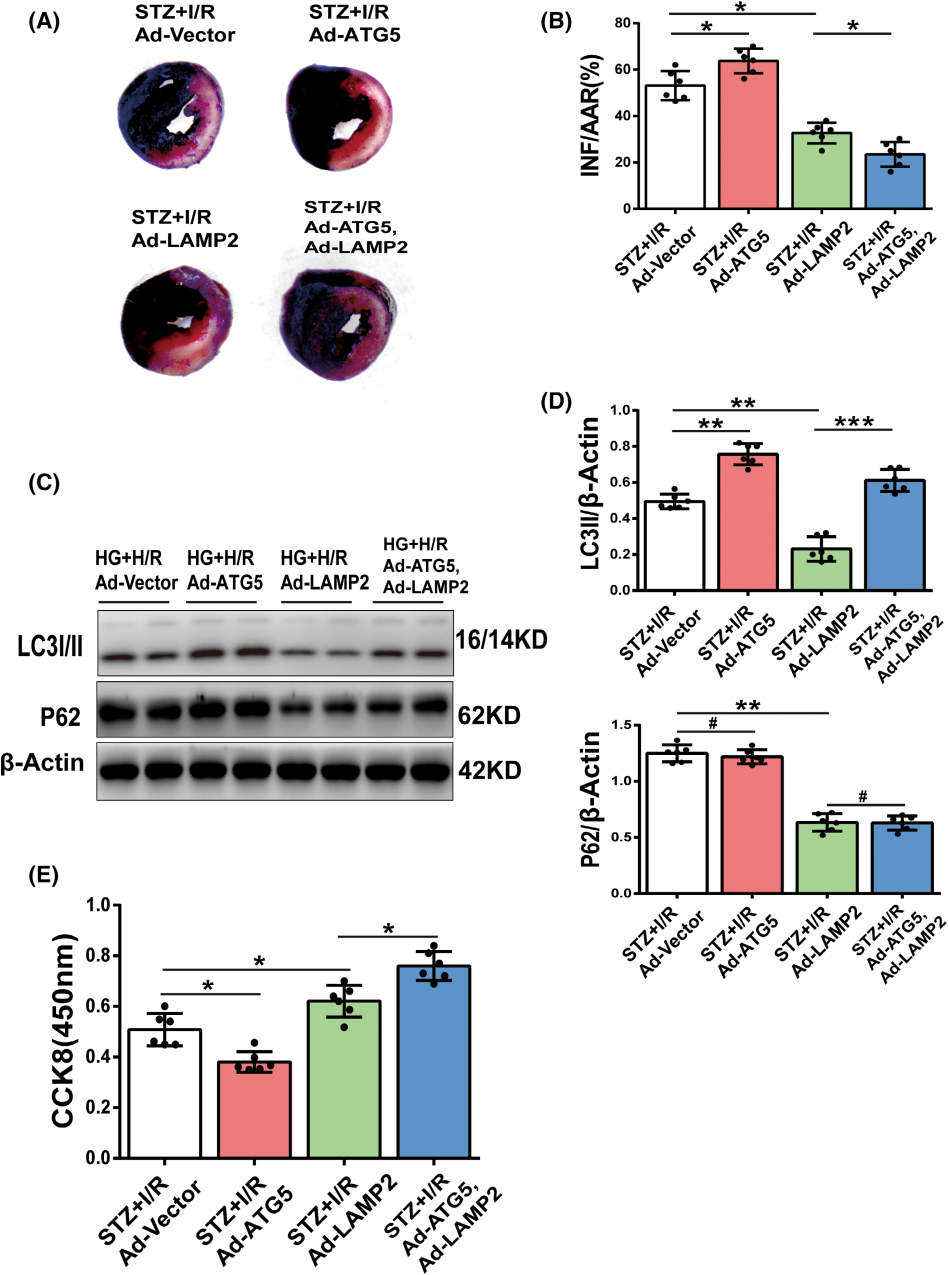
Effect of overexpression of Atg5 and LAMP2 on diabetic MIRI. (A,B) Diabetic mice were transfected with ad‐vector, ad‐ATG5 or ad‐LAMP2 by injection to anterior wall, and after 3 days subjected to I/R. Myocardial infarction size was shown by Evans blue‐TTC staining and quantitative analyses. (C,D) Cultured cardiomyocytes under HG condition were transfected with ad‐GFP, ad‐ATG5 or ad‐LAMP2 for 72 h and then subjected to H/R. LC3II and P62 expression by Western blotting and quantitative analyses. (E) Cardiomyocyte viability by CCK8. (*n* = 6 per group). *p < 0.05; **p < 0.01; ***p < 0.001; ^#^ *p* > 0.05.

To confirm the roles of LAMP2 and Atg5 in diabetic MIRI, the myocardium of wild‐type mice was injected with Ad‐vector, Ad‐LAMP2 and Ad‐ATG5 in the area of the anterior descending branch 3 days before I/R surgery. Transfection with ad‐LAMP2 mitigated the area of myocardial infarction from 57% to 31%, and co‐transfection with Ad‐LAMP2 and Ad‐ATG5 gained more protection to approximately 21%. Nevertheless, Atg5 overexpression in cardiomyocytes extended the infarction to 62% (Figure 6A,B).

In vitro, we transfected or co‐transfected Ad‐vector, Ad‐LAMP2 and Ad‐ATG5 into cardiomyocytes before they were subjected to HG + H/R conditions. Ad‐ATG5 and ad‐LAMP2 co‐transfection resulted in an elevation of LC3II expression, decreased P62 expression and regained cellular viability compared to the Ad‐vector transfection group, suggesting the restoration of autophagic flux and corresponding protection against diabetic MIRI (Figure 6C‐E). LAMP2 overexpression decreased the expression levels of both LC3II and P62 and mildly increased cellular viability under HG + H/R stress, indicating that recovering LAMP2 facilitates autophagosome‐lysosome fusion and degradation and benefits cellular survival (Figure 6C‐E). However, cellular viability deteriorated after Atg5 overexpression with upregulated LC3II expression and similar P62 levels compared to the Ad‐vector transfection group (Figure 6C‐E). We speculated that promoting autophagosome formation without repairing clearance would be harmful. These results demonstrate that recovered LAMP2 and Atg5 expression restores autophagic flux and protects the diabetic heart from I/R injury.

## 4 DISCUSSION

In this study, we illustrate that cardiomyocyte autophagic flux impairment is exaggerated in diabetic I/R injury compared to nondiabetic I/R injury, contributing to worse infarction size and heart function. Calpain‐1/2 additive upregulation and activation during diabetic I/R is responsible for the excessive autophagic flux impairment. The degradation of Atg5 and LAMP2 is associated with calpain activation, and inhibiting calpain activity or co‐overexpression of LAMP2 and Atg5 could restore autophagic flux and reduce myocardial injury in diabetic I/R. However, overexpression of Atg5 alone appears to be harmful.

Adaptive enhanced autophagic flux enables cardiomyocytes to survive under energy stress, such as I/R, by maintaining ATP supply, providing amino acids for protein synthesis and eliminating damaged mitochondria, and autophagic flux impairment can result in excessive ROS, harmful protein fragments and dysfunctional organelles, which can cause cell disruption and death^8^ and nullify the effects of pre‐ and postconditioning.^20^ We found exaggerated autophagic flux impairment in experimental diabetic I/R hearts, characterized by less autophagosome formation, fewer autolysosomes and accumulated damaged mitochondria compared to nondiabetic I/R hearts. Rapamycin pretreatment or calpain inhibition attenuated diabetic MIRI by enhancing autophagic flux, but the autophagic flux inhibitors 3‐MA and CQ worsened injury. In summary, exaggerated impairment of autophagic flux is involved in the vulnerability of experimental diabetic hearts to MIRI.

Calpain‐1 and calpain‐2 are ubiquitous in cardiomyocytes and can be activated by an increase in calcium (Ca2+) concentration, or by mitogen‐activated protein kinase, protein kinase C, protein kinase A and NADPH oxidase.^12^ Previous studies on diabetic cardiomyopathy have demonstrated that mitochondrial calpain‐1 upregulation induces the generation of mitochondrial ROS by cleavage of ATP synthase‐α,^21^ and cytosolic calpain‐1 activation is associated with cardiomyocyte apoptosis by inhibition of Na+/K+ ATPase activity with high‐glucose stimulation.^22^ In this study, the protein expression and activity of calpain‐1 were upregulated in diabetic mouse hearts. When subjected to I/R, diabetic hearts present a much higher calpain‐1 and calpain‐2 expression and activity than nondiabetic I/R hearts, which may be due to the enhancement of Ca2+ concentration and NADPH oxidase activity, and a decrease in the Ca2+ requirement for activation.^23^

Atg5 is an essential protein for the Atg12‐Atg5 complex and autophagosome membrane elongation.^7^ Inhibition of Atg5 in HL‐1 cells during I/R reduces autophagosomes and increases I/R injury.^24^ LAMP2 plays a crucial role in lysosome stability and autophagosome‐lysosome fusion.^25^ Lamp2‐knockout mice show hypertrophic cardiomyopathy with accumulated autophagic vacuoles. Overexpression of LAMP2 in cardiomyocytes during hypoxia alleviates lysosomal membrane permeabilization and facilitates autophagic flux.^26^ In this study, the asynchronous mRNA and protein expression profiles of Atg5 and LAMP2 indicate the degradation of the two proteins in diabetic I/R hearts. We detected calpain‐dependent downregulation of Atg5 and LAMP2 and the presence of cleaved fragments in diabetic I/R hearts, and calpain inhibition or co‐overexpression of Atg5 and LAMP2 restored autophagic flux and rescued diabetic MIRI. Cleaved fragments of Atg5 and LAMP2 are also present in diabetic hearts and nondiabetic I/R hearts, but are most pronounced in diabetic I/R hearts, which coincides with calpain activity. These data suggest that highly activated calpains and calpain‐mediated downregulation of Atg5 and LAMP2 are associated with exaggerated autophagic flux impairment of diabetic MIRI. The impact of diabetes on myocardial autophagy is complex. Most studies have demonstrated lower autophagy activity in cardiomyocytes of type 1 and type 2 diabetes due to activated mammalian target of rapamycin(mTOR) and inhibited AMPK and transcription factor EB(TFEB) signalling in diabetic hearts.^27^, ^28^ However, the effects of autophagy regulation are divergent. Although some drugs provide protection from diabetic MIRI through AMPK‐mediated autophagy upregulation.^29^, ^30^ overexpression of Beclin1 and Atg7 restores autophagy but worsens high glucose injury.^27^ In our study, we also found that supplementation with Atg5 alone aggravates diabetic MIRI. We suppose there are two possible explanations: first, abundant Atg5 enables autophagosome formation but has no effect on subsequent fusion and degradation, leading to autophagosome accumulation and autophagic death; second, the cleaved fragment of Atg5 is pro‐apoptotic in the presence of calpains.^15^

Although our present study focused on calpain‐mediated autophagy protein alterations, there are other mechanisms that may be involved in defective autophagic flux in diabetic MIRI. For instance, AMPK is downregulated in diabetes and the phosphorylation of AMPK is further suppressed during I/R, which would also aggravate autophagic flux impairment during diabetic I/R.^5^ Therefore, we suppose that the combination of an AMPK activator and a calpain inhibitor would be more protective against diabetic MIRI.

In conclusion, our study demonstrated that the vulnerability of the diabetic mouse heart to I/R injury is due, at least in part, to highly activated calpains that exaggerate autophagic flux impairment by cleaving Atg5 and LAMP2. Targeting autophagic flux and calpain activity could be a promising strategy to improve heart function in diabetic patients with heart revascularization.

## AUTHOR CONTRIBUTIONS

**Min Yu:** Conceptualization (equal); funding acquisition (lead); resources (equal); visualization (equal); writing – original draft (equal); writing – review and editing (equal). **Lichun Guan:** Conceptualization (equal); data curation (equal); formal analysis (equal); investigation (equal); methodology (equal); project administration (equal); resources (equal); visualization (equal); writing – original draft (equal); writing – review and editing (equal). **Ziqin Yu:** Formal analysis (equal); methodology (equal). **Zhimei Che:** Data curation (supporting); formal analysis (supporting); methodology (supporting); resources (supporting). **Hang Zhang:** Data curation (supporting); investigation (supporting). **Yong Yu:** Methodology (supporting); project administration (supporting). **Dicheng Yang:** Funding acquisition (supporting); resources (supporting). **Dewei Qian:** Data curation (supporting). **Ruizhen Chen:** Funding acquisition (supporting); investigation (supporting); methodology (supporting); resources (equal); supervision (equal).

## FUNDING INFORMATION

National Natural Science Foundation of China: 81770411, 81,300,094.

## CONFLICT OF INTEREST

None.

## Supporting information


FigureS1

FigureS2

## Data Availability

The data generated or analysed during this study are available from the corresponding author upon reasonable request.
