# Supplementary figures and images for: Experimental diabetes exacerbates autophagic flux impairment during myocardial I/R injury through calpain‐mediated cleavage of Atg5/LAMP2
Source: J Cell Mol Med. 2022 Dec 23;27(2):232–45. doi: 10.1111/jcmm.17642 (PMC9843523; doi:10.1111/jcmm.17642)

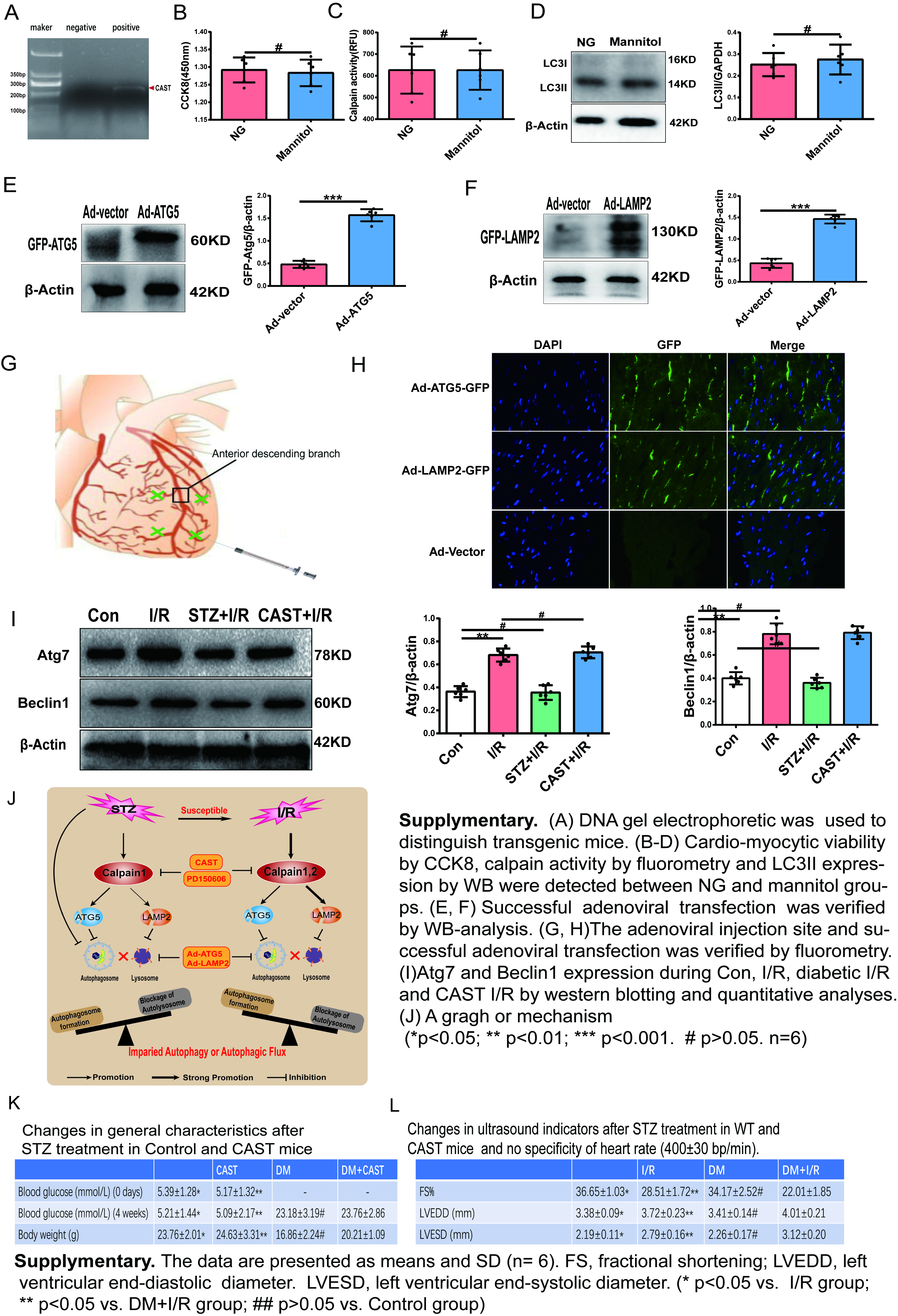

Supplement: Supplementary file 1 — FigureS1 [file JCMM-27-232-s001.tif]

Con

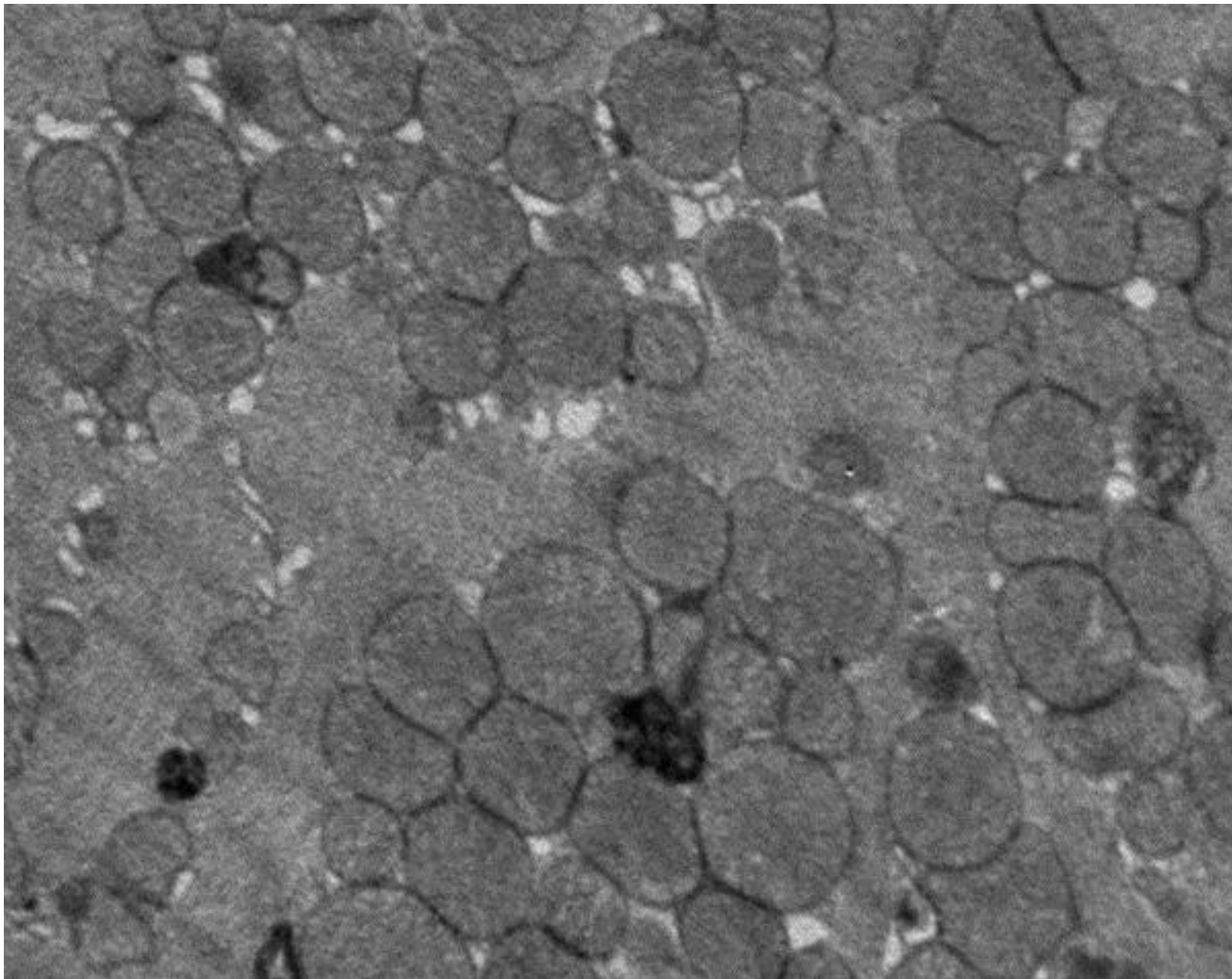

STZ

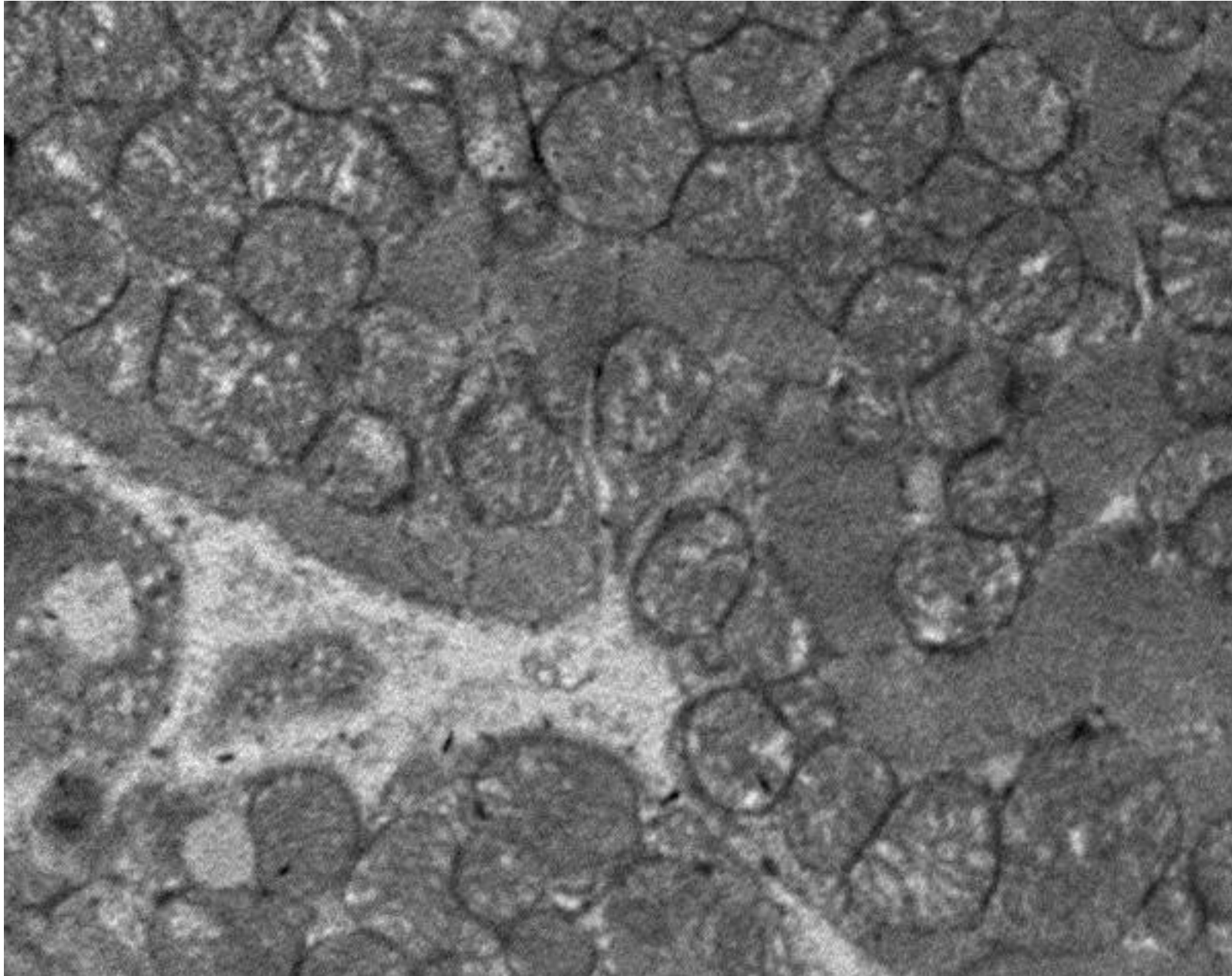

I/R

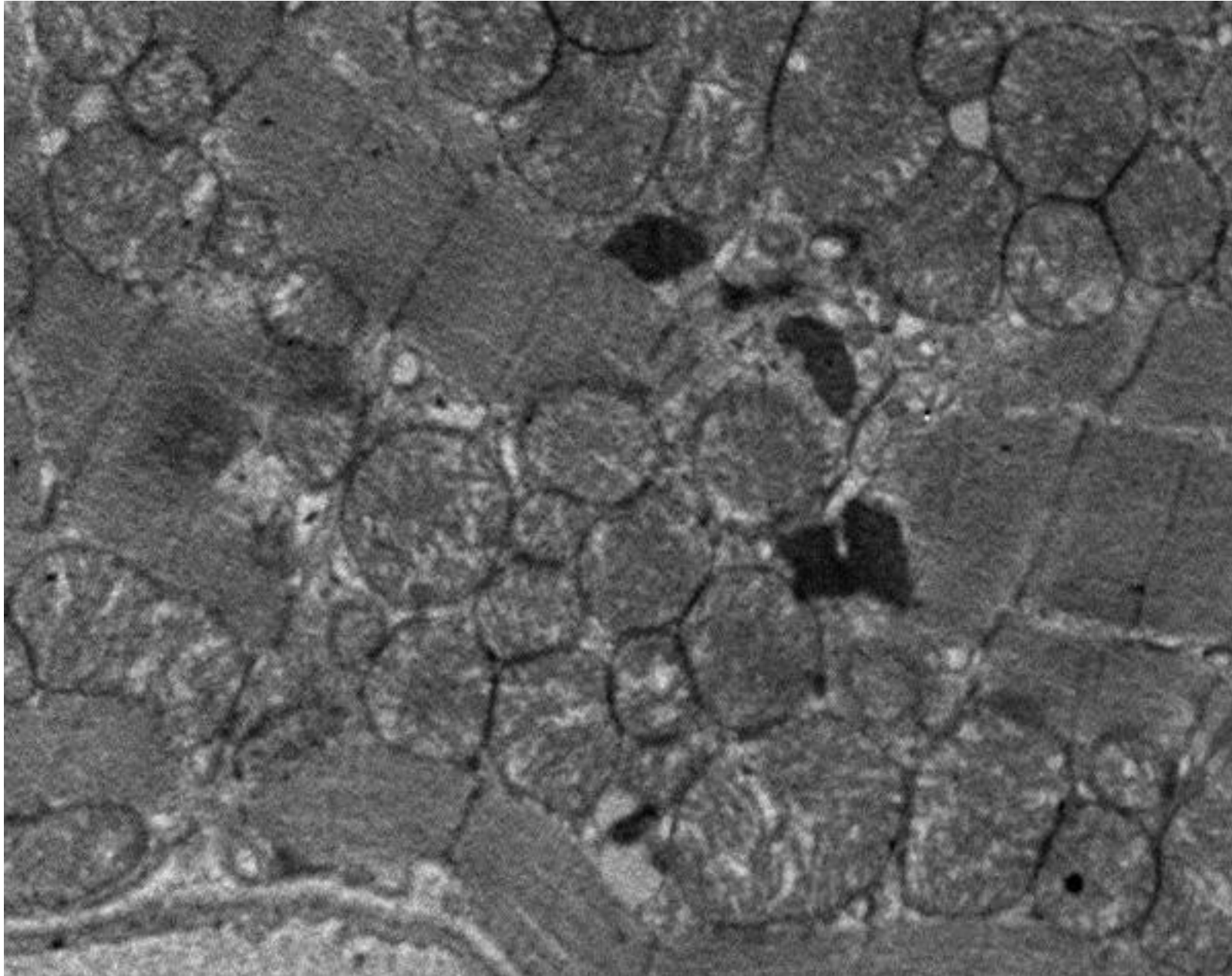

I/R+STZ

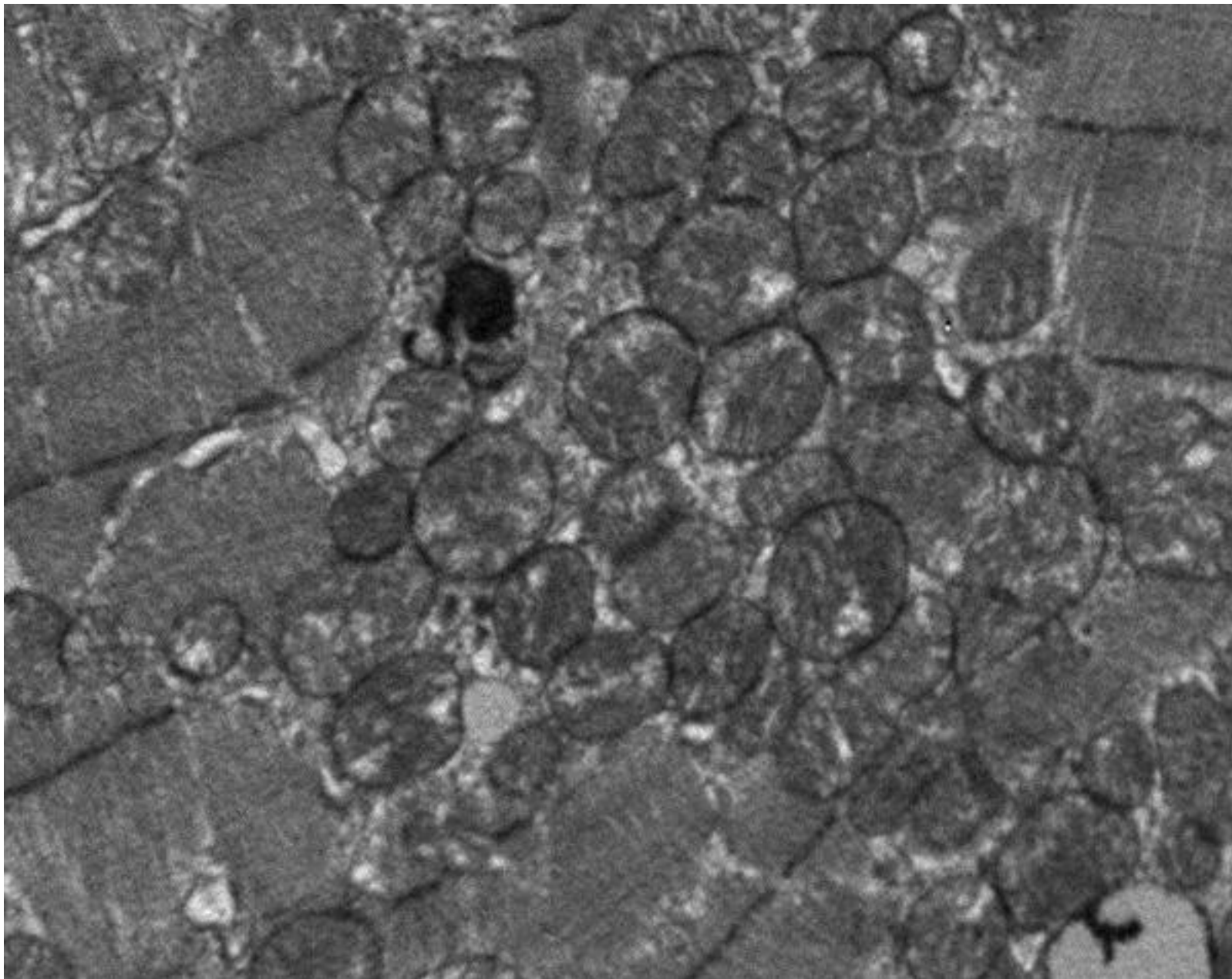

Supplement: Supplementary file 2 — FigureS2 [file JCMM-27-232-s002.pdf]
